# The safety and effectiveness of genetically corrected iPSCs derived from β-thalassaemia patients in nonmyeloablative β-thalassaemic mice

**DOI:** 10.1186/s13287-020-01765-w

**Published:** 2020-07-16

**Authors:** Yexing Xian, Yingjun Xie, Bing Song, Zhanhui Ou, Shuming Ouyang, Yuhuan Xie, Yi Yang, Zeyu Xiong, Haoxian Li, Xiaofang Sun

**Affiliations:** 1 Key Laboratory for Major Obstetric Diseases of Guangdong Province, Key Laboratory of Reproduction and Genetics of Guangdong Higher Education Institutes, Guangzhou Regenerative Medicine and Health Guangdong Laboratory, The Third Affiliated Hospital of Guangzhou Medical University, Guangzhou, 510150 Guangdong Province China; 2 Guangzhou Regenerative Medicine and Health Guangdong Laboratory, Guangzhou, 510005 Guangdong Province China

**Keywords:** iPSCs, β-Thalassaemia, Transplantation

## Abstract

**Background:**

β-Thalassaemia is a clinically common cause of hereditary haemolytic anaemia stemming from mutations in important functional regions of the β-globin gene. The rapid development of gene editing technology and induced pluripotent stem cell (iPSC)-derived haematopoietic stem cell (HSC) transplantation has provided new methods for curing this disease.

**Methods:**

Genetically corrected β-thalassaemia (homozygous 41/42 deletion) iPSCs that were previously established in our laboratory were induced to differentiate into HSCs, which were transplanted into a mouse model of IVS2–654 β-thalassaemia (B6;129P2-*Hbb*^*tm2Unc*^/J mice) after administration of an appropriate nonmyeloablative conditioning regimen. We also investigated the safety of this method by detecting the incidence of tumour formation in these mice after transplantation.

**Results:**

The combination of 25 mg/kg busulfan and 50 mg/(kg day) cyclophosphamide is an ideal nonmyeloablative protocol before transplantation. Genetically corrected β-thalassaemic HSCs survived and differentiated in nonmyeloablated thalassaemia mice. No tumour formation was observed in the mice for 10 weeks after transplantation.

**Conclusion:**

Our study provides evidence that the transplantation of genetically corrected, patient-specific iPSCs could be used to cure genetic diseases, such as β-thalassaemia major.

## Introduction

β-Thalassaemia is one of the most common autosomal recessive disorders worldwide [1]. Due to the presence of over 200 different mutations in important functional regions of the β-globin gene [2], β-thalassaemia patients have a defect in β-haemoglobin formation, and their clinical manifestations vary from minor symptoms to severe anaemia. Regardless of the genotype, the classification and severity of this disease are based primarily on haemoglobin (HB) levels and clinical tolerability. This genetically inherited disease, which is prevalent throughout southern China, has threatened millions of people’s lives for decades, but no effective treatments are available. Patients with severe β-thalassaemia need regular red blood cell injections to survive [3]; without treatment, these patients will die within the first 5 years of life, and even with transfusions, only 50–65% of patients in high-income countries survive to 35 years of age [4–6].

Gene therapy with haematopoietic stem/progenitor cells (HSPCs) has been clinically used for the treatment of β-thalassaemia, and although its efficacy and safety are still under evaluation, it has shown promising therapeutic effects [7–9]. Induced pluripotent stem cell (iPSC) technology emerged in 2006 [10] and has developed rapidly in recent years, providing an ideal research tool and treatment strategy in regenerative medicine. In addition, obtaining iPSCs from patients with the same genetic background represents a solution for the ethical and immune rejection problems presented by the use of embryonic stem cells (ESCs). Moreover, similar to haematopoietic stem cells (HSCs), iPSCs can differentiate into the three germ layers (endoderm, mesoderm and ectoderm) in vitro [11–13]. HSCs can be obtained from embryoid bodies formed in the presence of haematopoietic cytokines or from cocultures of iPSCs with stromal cells [14]. Recently, many researchers have attempted to determine the curative effect of iPSC transplantation on haematological disease by using total body irradiation (TBI) or radiation combined with myeloablative drugs as pretreatment before transplantation, but the observed effects were not those expected [15, 16].

Therefore, in this study, iPSCs derived from patients with β-thalassaemia (homozygous 41/42 mutation) established by our laboratory [17] were used to generate HSCs, which were transplanted into B6;129P2-*Hbb*^*tm2Unc*^/J (ivs2-654) mice, a model of IVS2–654 β-thalassaemia [18]; we observed whether these HSCs can survive, home and differentiate in vivo and further evaluated their safety and effectiveness. In addition, we investigated the use of busulfan combined with cyclophosphamide as a nonmyeloablative conditioning regimen prior to stem cell transplantation to more closely approximate clinical treatment. Our results demonstrated that genetically corrected, iPSC-derived HSCs can differentiate in vivo and produce human β-globin in a mouse model, and the mice that received transplants lived for over 10 weeks without tumour formation. These results have important implications for the future personalized treatment of β-thalassaemia.

## Materials and methods

### Experimental animals

We used the B6;129P2-*Hbb*^*tm2Unc*^/J thalassaemia mouse model purchased from The Jackson Laboratory (Stock No: 003250). Animals were housed in an SPF animal room at the Guangdong Province Experimental Animal Centre for Medical Laboratory (experimental animal licence number: SYXK (Guangdong) 2013-0002) under the following conditions: temperature, 20~26 °C; humidity, 40~70%; and 10-h:14-h day/night cycle. The housing conditions remained stable to ensure the reliability of the experimental results. The heterozygous mouse model (Hbb^th-4^/Hbb^+^) carries one allele with the human β-IVS-2-654 mutation and one normal mouse β-globin allele and exhibits a mild β-thalassaemia phenotype, microcytosis and other erythrocyte morphologies reflecting reduced mouse β-globin levels and no human β-globin [18]. Experiments were performed using 8–10-week-old healthy female mice weighing 20 ± 2 g and positive for β-thalassaemia.

### Determination of the nonmyeloablative drug dosage

Experimental grouping and treatment: The mice were randomly divided into 4 groups of 7 mice each. The mice in the experimental groups (groups 22, 25, 28 and 35) were intraperitoneally injected with busulfan (Patheon Manufacturing Services, LLC) at 22 mg/(kg day), 25 mg/(kg day), 28 mg/(kg day) and 35 mg/(kg day), respectively. Mice in all the experimental groups were given the same dose of 50 mg/(kg day) cyclophosphamide (Baxter Oncology GmbH) on the 5th or 6th day. Mice in the normal control group were injected intraperitoneally with 0.9% sodium chloride. After 7 days, the white blood cells (WBCs) were subjected to routine blood tests every other day. Bone marrow (BM) pathology was assessed in two mice per group on day 11 (D11).

### Feeder-free culture of hESCs and iPSCs

Human ESCs (hESCs; H1), niPSCs (iPSCs from normal human blood), piPSCs (β-iPS-41/42 cell line generated using cells from a β-thalassaemia patient homozygous for the CD41/42 (-CTTT) HBB mutation and infected with Sendai virus) and ciPSCs (genetically corrected iPSCs previously homozygous for the 41/42 deletion) were established in the Third Affiliated Hospital of Guangzhou Medical University. Cell lines were cultured using mTeSR™1 medium (STEMCELL Technologies) and Matrigel (BD Biosciences) in a feeder-independent system according to the manufacturer’s instructions.

### Haematopoietic differentiation

OP9 cells were placed onto 0.1% gelatine-coated 100-mm culture dishes in α-MEM (Invitrogen) containing 10% foetal bovine serum (FBS) (BI) and 50 μg/ml penicillin/streptomycin (Gibco). When the OP9 cells reached confluence, half of the medium was exchanged every other day. Undifferentiated hESCs and iPSCs were harvested after OP9 cells were cultured for 5–6 days at confluence. Undifferentiated iPSCs or ESCs were washed with DMEM/F12 and then digested with dispase. The cells were collected by scraping, pipetted into tubes and observed until they sedimented naturally. The supernatant was discarded, and an appropriate amount of culture medium was added to resuspend the cells. Next, 1.5 × 10^6^ stem cells were added to OP9 cells in 100-mm dishes with α-MEM supplemented with 10% FBS, 100 μM monothioglycerol (MTG; Sigma), 20 ng/ml IL-3 (PeproTech), 40 ng/ml SCF (PeproTech), 20 ng/ml VEGF (PeproTech), 20 ng/ml BMP4 (PeproTech) and 20 ng/ml Flt3 (PeproTech). The coculture system was maintained for up to 12 days in an incubator at 37 °C in 5% CO_2_, and half of the medium was exchanged every other day.

### Colony forming unit (CFU) assay

CD34^+^ cells were sorted by flow cytometry, and approximately 5–10 × 10^4^ single cells were suspended in MethoCult H4434 (STEMCELL Technologies) and then placed in an incubator at 37° and 5% CO_2_ for at least 14 days. Shaking of the culture dish during cultivation was avoided to prevent disrupting the colonization pattern. Images of the typical morphology of different types of colonies were obtained after 14 days of culture.

### Collection of CD34^+^ cells

CD34^+^ cells from iPSC/OP9 and hESC/OP9 cell cocultures and human umbilical cord blood were collected, washed with magnetic-activated cell sorting (MACS) buffer (PBS with 2% FBS) and incubated with FcR Blocking Reagent and CD34 MicroBeads (Miltenyi Biotec) for 30 min at 4 °C. Then, the CD34^+^ cells were magnetically separated on MS columns according to the manufacturer’s instructions.

### BM transplantation

Mice in each group consumed sterile water supplemented with gentamicin (320 mg/l) starting 5 days before drug treatment and continuing for 3 weeks after transplantation to avoid intestinal infections. The optimal dose of busulfan was intraperitoneally injected on D1–4, and 50 mg/(kg day) cyclophosphamide was injected intraperitoneally on D5–6. On D7, the mice were anaesthetized with isoflurane, and an insulin syringe was rotated parallel to the bottom of the left femur of the mouse for insertion into the femoral marrow cavity. When there was no resistance to the puncture, the syringe was inserted into the marrow cavity.

The mice received nonmyeloablative treatment and then were randomly divided into 6 groups: the α-MEM, piPSC, ciPSC, niPSC, UCBC and hESC groups. For cell transplantation, 20 μl of α-MEM containing 5 × 10^5^ CD34^+^ cells that were differentiated or isolated from each group of stem cells was inserted into the BM cavity. The mice were warmed with a warmer before being awakened. Starting on the next day, 100 μl of 60 μg/ml recombinant human granulocyte colony-stimulating factor (rhG-CSF) and 100 μl of 100 IU/ml recombinant human erythropoietin (rhEPO) were intraperitoneally injected into each mouse that underwent transplantation. The negative control group was injected with 200 μl of normal saline. The treatments and control were administered 6 times on an every-other-day schedule.

### Routine blood tests

Approximately 30-μl blood samples were collected from mice. Routine tests were performed every week using the Sysmex XN-9000 system according to the manufacturer’s instructions for dilution mode.

### Flow cytometry

hESC/OP9 and iPSC/OP9 cell cocultures were harvested and washed with FACS buffer (PBS with 2% FBS). Cells were analysed for HSC differentiation efficiency using an anti-human CD34-PE-Cy7 monoclonal antibody (BD). Mouse peripheral blood cells and femur BM cells were collected to analyse the human cell ratios. The cells were treated with erythrocyte lysate (E Bioscience), washed with FACS buffer and stained with the following monoclonal antibodies from BD Biosciences: anti-human CD3-FITC, anti-human CD8-PE-Cy7, anti-human CD31-PE, anti-human CD34-PE-Cy7, anti-human CD43-FITC, anti-human CD45-APC and anti-human CD71-PE. Staining of erythrocytes with anti-human CD235-APC monoclonal antibody (BD Biosciences) does not require lysis. Finally, the cells were resuspended in 200 μl of FACS buffer and analysed by flow cytometry on a weekly basis for 10 weeks after transplantation.

### Pathological examination

All mice were sacrificed 10 weeks after transplantation. Blood and BM cells from both femurs were collected for flow cytometry analysis. The liver, lungs, kidneys and BM were collected for pathological examination.

### Statistical analysis

Data are expressed as the mean ± standard error of the mean (SEM) after comparison by one-way analysis of variance (ANOVA). Significant differences between groups identified by ANOVA were assessed by the post hoc LSD test (SPSS version 13.0 for Windows). Differences with *P* < 0.05 were considered statistically significant.

## Results

### Peripheral blood results in mice after nonmyeloablative pretreatment

**Fig. 1:**
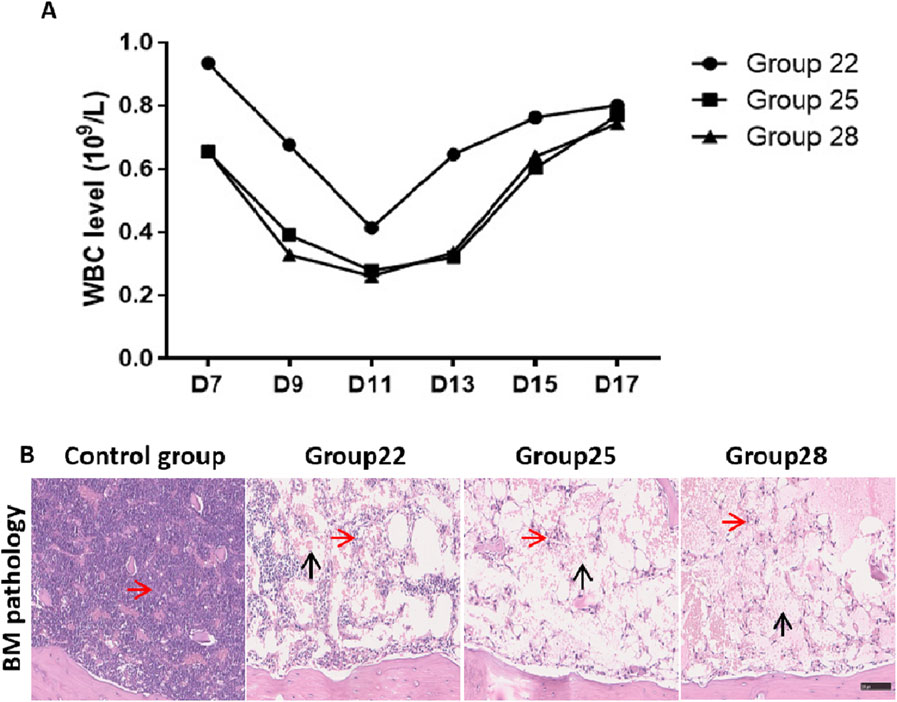
WBC evaluation of β-thalassaemic mice in each group after treatment with different doses of nonmyeloablative drugs. **a** Different doses of busulfan in each group. **b** BM pathology in β-thalassaemic mice after nonmyeloablative treatment. Bone tissue from groups 25 and 28 showed only a few haematopoietic cells (red arrow). The remaining cavities were filled with vacuole structures (black arrow); scale bar, 20 μm

In our study, WBCs from mice in each experimental group showed different degrees of decline after drug treatment (Fig. 1a). All mice in group 35 died during drug treatment; therefore, leukocytes and BM pathology were not assessed in this group. The WBC counts in groups 22, 25 and 28 reached a minimum on D11 (0.41 × 10^9^/l, 0.27 × 10^9^/l and 0.26 × 10^9^/l, respectively) and then began to recover. The WBC count was lower on D9 in group 22 than in the other groups (0.67 vs 0.39 and 0.32) but recovered more quickly in group 22 than in the other two groups (D13: 0.64 vs 0.32 and 0.33). In terms of the degree of decline and recovery of WBCs, both were lower in groups 25 and 28 than in group 22. The greater the degree of preparation was, the easier it was for the mice to accept the exogenous cell implantation. Although groups 28 and 25 were similar in terms of WBC profiles, group 28 had significantly greater mortality than group 25 due to the high dose of preparative drugs. Therefore, we determined that the dose administered to group 25 was the most appropriate.

In the BM pathology results on D11 (Fig. 1b), purple represents HSCs. With the increase in busulfan dose, the number of HSCs in the BM cavity decreased. However, a few HSCs were observed, and the remaining cavities were filled with vacuolar structures. Our results showed that the WBC count in group 25 was relatively stable, and mortality was low. Considering these results, 25 mg/kg busulfan was chosen as the best nonmyeloablative treatment for subsequent experiments.

### Haematopoietic differentiation in vitro

**Fig. 2:**
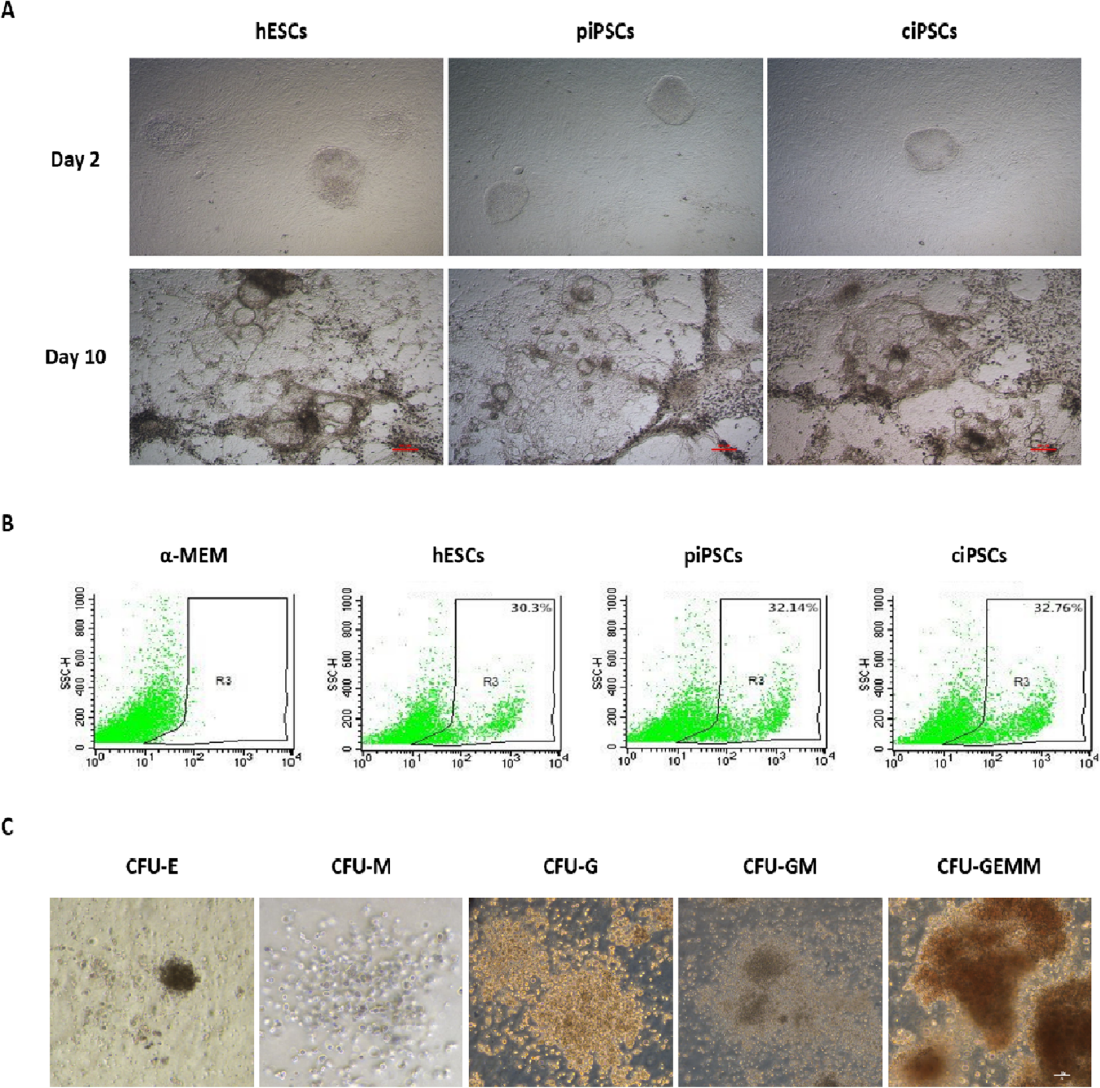
Morphology of iPSCs and ESCs in the OP9 coculture system and CFU assays performed with CD34^+^ cells from different groups. **a** Images of the typical morphology of iPSCs and ESCs in the OP9 coculture system used to induce haematopoietic differentiation at D2 and D10; scale bar, 100 μm. **b** Flow cytometry analysis of human CD34^+^ cell proportions in the four groups (α-MEM, hESC, piPSC and ciPSC groups) after coculture with OP9 cells. **c** Morphology of colonies of CD34^+^ haematopoietic progenitors derived from iPSCs cocultured with OP9 cells; scale bar, 50 μm. HSCs induced differentiation into erythroid cells (E), macrophages (M), granulocytes (G), granulocytes/macrophages (GMs) and granulocytes/erythrocytes/monocytes/megakaryocytes (GEMMs)

To determine and compare haematopoietic and erythroid differentiation abilities, piPSC, ciPSC and hESC lines were each cocultured with OP9 stromal cells using previously established protocols [19]. Two and 10 days after coculture, the morphology of these cells indicated similar differentiation processes (Fig. 2a). It is noteworthy that both uncorrected (piPSCs) and genetically corrected (ciPSCs) iPSCs cocultured with stromal cells had a similar ability to differentiate and produce HSCs. On D12, cells expressing the haematopoietic cell marker CD34 were analysed by flow cytometry. Our results revealed a similar percentage of CD34^+^ cells derived from all cell lines: hESCs, 30.3%; piPSCs, 32.14%; and ciPSCs, 32.76% (Fig. 2b).

These results indicated that both uncorrected and genetically corrected iPSCs are capable of successful haematopoietic differentiation in vitro, and higher efficiency was observed with our combination of cytokines.

Next, we used haematopoietic CFU assays to further assess the haematopoietic differentiation ability of ESCs and iPSCs in vitro. Our results showed that CD34^+^ haematopoietic progenitors from the OP9 coculture system formed colonies of various cell types, including erythroid cells, macrophages, and granulocytes, and multipotent colonies containing granulocyte, macrophage and/or megakaryocyte elements (CFU-E, CFU-M, CFU-GM and CFU-GEMM, respectively) (Fig. 2c).

### Haematopoietic differentiation in vivo

Next, an in vivo haematopoietic differentiation assay was performed with B6;129P2-*Hbb*^*tm2Unc*^/J mice as the experimental model. Heterozygous mice are considered an ideal model because they exhibit symptoms consistent with a moderate form of β-thalassaemia, including haemolytic anaemia, microcytosis and other erythrocyte morphology reflecting a reduction in β-globin and the absence of human β-globin. Therefore, we used this mouse model to observe the haematopoietic differentiation of ciPSCs in vivo. For these experiments, 10 × 10^5^ CD34^+^ HSCs were enriched by MACS and transplanted into the left femur of drug-treated mice. Routine blood analyses were performed up to 5 weeks after transplantation to evaluate the recovery of haematopoietic function.

**Fig. 3:**
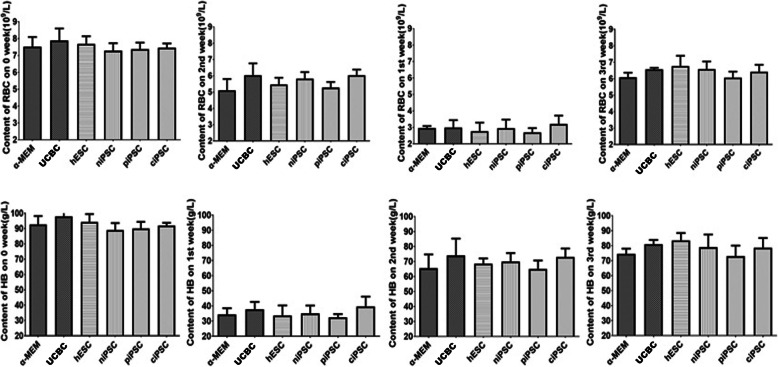
Changes in HB levels and RBC counts in the peripheral blood of β-thalassaemic mice in the four groups transplanted with α-MEM, UCBCs, hESCs, niPSCs, piPSCs or ciPSCs from week 0 to week 3 after transplantation. Error bars represent the S.D. (*n* = 3). HB, haemoglobin; RBC, red blood cell

Similar to the results of other studies [19], those of our study showed that the erythrocyte (RBC) count and HB levels in the six transplantation groups of mice were lower in the first week after transplantation than at baseline (3.6 × 10^12^/l and 100 g/l, respectively) (*P* < 0.05). Two weeks after transplantation, the HB level in each experimental group began to increase and was higher than that in the control group (Table 1). The RBC count in all groups of mice that underwent transplantation began to normalize (over 3.6 × 10^12^/l) in the second week after the transplant. In all groups of mice, HB levels increased slowly and remained below the normal baseline until the 5th week after transplantation (Fig. 3, Table 1). However, there were no significant differences between these groups in HB levels or RBC count at different weeks post transplantation (*P* > 0.05) (Fig. 3).

**Table 1:** Summary of routine blood test parameters of six groups of β-thalassaemic mice before and after transplantation from week 0 to week 5 after nonmyeloablative treatment

	**Weight (g)**	**RBC (10**^**12**^**/l)**	**HB (g/l)**	**HCT (%)**
**0 week**				
α-MEM group (*n* = 3)	19.30 ± 0.71	7.52 ± 0.58	92.67 ± 5.51	31.07 ± 2.37
UCBC group (*n* = 3)	18.77 ± 1.68	7.89 ± 0.71	98.00 ± 8.00	32.33 ± 3.55
hESC group (*n* = 3)	19.70 ± 0.90	7.69 ± 0.45	94.33 ± 5.13	31.77 ± 1.44
niPSC group (*n* = 3)	19.77 ± 0.68	7.28 ± 0.45	89.00 ± 4.58	30.43 ± 1.61
piPSC group (*n* = 3)	18.93 ± 1.24	7.38 ± 0.39	90.00 ± 4.36	31.13 ± 0.50
ciPSC group (*n* = 3)	20.00 ± 1.39	7.46 ± 0.25	92.00 ± 1.73	31.05 ± 0.78
**1 week**				
α-MEM group (*n* = 3)	20.80 ± 0.85	2.96 ± 0.13	34.33 ± 4.16	18.17 ± 3.84
UCBC group (*n* = 3)	20.60 ± 1.50	2.99 ± 0.45	37.67 ± 5.03	12.50 ± 2.36
hESC group (n = 3)	21.10 ± 0.72	2.77 ± 0.53	33.67 ± 6.66	11.63 ± 2.91
niPSC group (*n* = 3)	21.00 ± 0.36	2.95 ± 0.53	35.00 ± 5.29	7.77 ± 2.70
piPSC group (*n* = 3)	20.80 ± 0.70	2.70 ± 0.26	32.50 ± 2.12	14.25 ± 1.63
ciPSC group (*n* = 3)	20.93 ± 1.88	3.20 ± 0.52	39.67 ± 6.51	15.53 ± 4.45
**2 weeks**				
α-MEM group (*n* = 3)	21.55 ± 1.63	5.11 ± 0.69	65.50 ± 9.19	33.00 ± 12.02
UCBC group (*n* = 3)	21.90 ± 0.85	6.04 ± 0.73	74.00 ± 11.31	33.45 ± 7.00
hESC group (*n* = 3)	20.13 ± 2.53	5.47 ± 0.42	68.50 ± 3.54	22.13 ± 13.86
niPSC group (*n* = 3)	19.17 ± 3.41	5.83 ± 0.40	70.00 ± 5.66	18.00 ± 20.38
piPSC group (*n* = 3)	22.45 ± 0.07	5.28 ± 0.35	65.00 ± 5.66	32.65 ± 2.76
ciPSC group (n = 3)	22.27 ± 2.16	6.03 ± 0.35	73.00 ± 5.66	25.30 ± 7.91
**3 weeks**				
α-MEM group (*n* = 3)	21.60 ± 0.99	6.08 ± 0.28	74.50 ± 3.54	33.30 ± 0.57
UCBC group (*n* = 3)	22.30 ± 0.85	6.57 ± 0.09	81.00 ± 2.83	28.35 ± 0.21
hESC group (*n* = 3)	20.70 ± 1.13	6.77 ± 0.62	83.50 ± 4.95	30.85 ± 3.18
niPSC group (*n* = 3)	21.00 ± 1.27	6.58 ± 0.47	79.00 ± 8.49	32.45 ± 3.04
piPSC group (*n* = 3)	20.90 ± 0.14	6.06 ± 0.37	73.00 ± 7.07	30.40 ± 2.55
ciPSC group (*n* = 3)	21.50 ± 1.37	6.42 ± 0.42	78.67 ± 6.43	30.20 ± 1.08
**4 weeks**				
α-MEM group (*n* = 3)	21.40 ± 1.70	6.61 ± 0.46	82.50 ± 3.54	29.60 ± 0.14
UCBC group (*n* = 3)	22.80 ± 1.56	7.09 ± 0.64	86.50 ± 4.95	30.40 ± 3.11
hESC group (*n* = 3)	21.05 ± 0.78	6.97 ± 0.70	87.50 ± 4.95	31.30 ± 2.40
niPSC group (*n* = 3)	21.20 ± 1.41	6.97 ± 1.52	85.50 ± 17.68	31.05 ± 7.00
piPSC group (*n* = 3)	20.05 ± 0.21	6.92 ± 0.28	85.00 ± 5.66	30.00 ± 0.28
ciPSC group (*n* = 3)	21.17 ± 1.61	6.42 ± 0.17	79.33 ± 2.31	29.30 ± 1.61
**5 weeks**				
α-MEM group (*n* = 3)	23.95 ± 1.06	6.78 ± 0.50	83.00 ± 5.66	30.10 ± 0.14
UCBC group (*n* = 3)	23.10 ± 0.07	6.91 ± 0.21	84.00 ± 1.41	28.65 ± 1.20
hESC group (*n* = 3)	23.45 ± 1.34	6.51 ± 0.08	78.50 ± 0.71	28.00 ± 0.71
niPSC group (*n* = 3)	23.55 ± 1.34	6.91 ± 1.03	86.50 ± 14.85	30.00 ± 4.95
piPSC group (*n* = 3)	22.25 ± 0.21	6.39 ± 0.22	79.00 ± 4.24	27.00 ± 0.57
ciPSC group (*n* = 3)	23.87 ± 1.66	6.58 ± 0.79	81.33 ± 10.02	29.23 ± 2.41
	**MCV (fl)**	**MCH (pg)**	**MCHC (g/l)**	**RET%**
**0 week**				
α-MEM group (*n* = 3)	41.27 ± 0.35	12.33 ± 0.21	298.67 ± 4.93	14.64 ± 2.06
UCBC group (*n* = 3)	40.97 ± 1.01	12.43 ± 0.12	304.00 ± 9.17	12.99 ± 3.18
hESC group (*n* = 3)	41.33 ± 0.57	12.30 ± 0.17	296.67 ± 3.21	13.16 ± 4.13
niPSC group (*n* = 3)	41.77 ± 0.38	12.23 ± 0.21	292.33 ± 2.89	13.14 ± 3.37
piPSC group (*n* = 3)	42.30 ± 1.65	12.23 ± 0.12	289.00 ± 10.15	14.99 ± 6.27
ciPSC group (*n* = 3)	43.00 ± 1.51	12.33 ± 0.15	287.00 ± 13.00	18.13 ± 1.76
**1 week**				
α-MEM group (*n* = 3)	47.07 ± 3.75	12.67 ± 0.29	270.00 ± 20.52	8.18 ± 4.05
UCBC group (*n* = 3)	41.60 ± 1.71	12.60 ± 0.50	304.00 ± 22.61	4.00 ± 0.64
hESC group (*n* = 3)	41.67 ± 3.00	12.17 ± 0.29	292.33 ± 18.58	3.88 ± 2.23
niPSC group (*n* = 3)	40.90 ± 3.15	12.43 ± 0.35	305.00 ± 22.61	3.85 ± 1.15
piPSC group (*n* = 3)	42.95 ± 1.34	12.50 ± 0.00	292.00 ± 8.49	4.42 ± 0.18
ciPSC group (*n* = 3)	47.83 ± 6.25	12.37 ± 0.06	261.67 ± 33.65	8.40 ± 5.98
**2 weeks**				
α-MEM group (*n* = 3)	50.45 ± 3.61	12.30 ± 0.71	243.50 ± 3.54	54.45 ± 4.88
UCBC group (*n* = 3)	49.70 ± 2.69	12.15 ± 0.21	245.00 ± 18.38	34.25 ± 19.16
hESC group (n = 3)	54.40 ± 1.35	12.07 ± 0.87	221.67 ± 10.21	40.75 ± 19.76
niPSC group (*n* = 3)	51.37 ± 3.86	11.30 ± 1.18	221.00 ± 31.95	30.13 ± 17.03
piPSC group (*n* = 3)	52.10 ± 4.95	12.50 ± 0.00	241.00 ± 22.63	56.30 ± 11.88
ciPSC group (*n* = 3)	54.10 ± 3.04	12.33 ± 0.15	227.67 ± 12.90	42.37 ± 15.43
**3 weeks**				
α-MEM group (*n* = 3)	47.95 ± 6.29	12.20 ± 0.14	256.50 ± 31.82	34.16 ± 28.91
UCBC group (*n* = 3)	43.15 ± 0.92	12.35 ± 0.21	285.50 ± 12.02	19.07 ± 1.07
hESC group (*n* = 3)	45.55 ± 0.49	12.35 ± 0.35	271.50 ± 12.02	19.72 ± 1.41
niPSC group (*n* = 3)	49.60 ± 8.20	12.00 ± 0.42	246.00 ± 49.50	30.98 ± 23.50
piPSC group (*n* = 3)	43.95 ± 1.63	12.45 ± 0.21	283.50 ± 14.85	20.51 ± 1.66
ciPSC group (*n* = 3)	47.23 ± 2.07	12.23 ± 0.29	259.67 ± 17.01	26.14 ± 6.27
**4 weeks**				
α-MEM group (*n* = 3)	44.95 ± 3.32	12.50 ± 0.28	278.50 ± 13.44	12.33 ± 0.04
UCBC group (*n* = 3)	42.85 ± 0.49	12.20 ± 0.42	285.00 ± 12.73	15.94 ± 5.49
hESC group (*n* = 3)	44.05 ± 0.21	12.35 ± 0.21	280.00 ± 5.66	14.27 ± 6.46
niPSC group (*n* = 3)	44.55 ± 0.35	12.30 ± 0.14	276.00 ± 5.66	11.85 ± 3.85
piPSC group (*n* = 3)	43.35 ± 1.34	12.30 ± 0.28	283.50 ± 16.26	16.03 ± 0.21
ciPSC group (*n* = 3)	45.63 ± 1.36	12.37 ± 0.06	271.33 ± 8.50	20.44 ± 8.75
**5 weeks**				
α-MEM group (*n* = 3)	43.55 ± 4.88	12.25 ± 0.07	276.00 ± 19.80	12.13 ± 2.93
UCBC group (*n* = 3)	41.45 ± 0.49	12.15 ± 0.21	293.50 ± 7.78	10.60 ± 2.10
hESC group (*n* = 3)	43.00 ± 0.57	12.05 ± 0.21	280.50 ± 9.19	10.25 ± 1.62
niPSC group (*n* = 3)	43.35 ± 0.64	12.50 ± 0.28	288.50 ± 2.12	29.83 ± 31.07
piPSC group (*n* = 3)	42.30 ± 0.57	12.35 ± 0.21	292.50 ± 9.19	12.98 ± 4.16
ciPSC group (*n* = 3)	44.57 ± 1.72	12.33 ± 0.06	277.67 ± 11.50	`13.38 ± 0.92

Values represent mean ± SD. *RBC* red blood cell count, *HB* haemoglobin, *HCT* haematocrit, *MCV* mean corpuscular volume, *MCH* mean corpuscular haemoglobin, *MCHC* mean corpuscular haemoglobin concentration, *RET* reticulocyte count

**Fig. 4:**
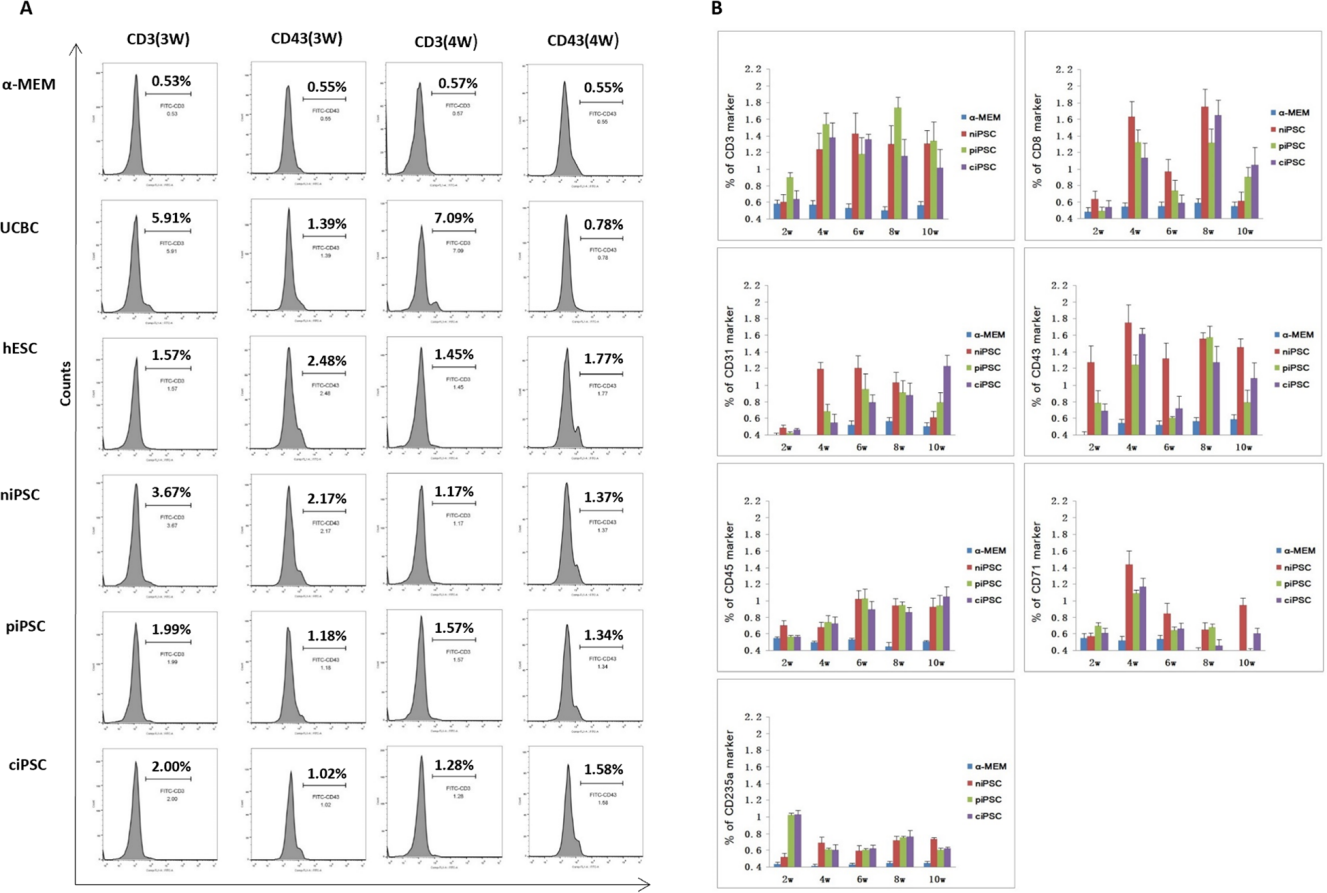
Haematopoietic differentiation of β-thalassaemia-iPSCs and genetically corrected iPSCs in the peripheral blood after transplantation. **a** Flow cytometry analysis of human CD3^+^ and CD43^+^ cell proportions among blood cells from the five groups of mice transplanted with α-MEM, UCBCs, hESCs, niPSCs, piPSCs or ciPSCs at weeks 3 and 4 after transplantation. **b** Consecutive test results for the α-MEM, piPSC, ciPSC and niPSC groups after transplantation; error bars represent the S.D. (*n* = 3)

Next, to determine the complete blood cell differentiation profile of the implanted cells, we analysed cluster of differentiation (CD) markers in blood using flow cytometry analysis. Human CD3 was present in 70–80% of normal human peripheral blood lymphocytes and 60–85% of thymocytes; human CD45 was present in all human leukocytes, including lymphocytes, monocytes, granulocytes, eosinophils and thymocytes; human CD71 was expressed on erythroid progenitors; human CD235a was expressed on human erythrocytes and erythrocyte precursor cells; human CD8 was expressed on most thymocytes; human CD31 was expressed on platelets, monocytes, granulocytes and most endothelial cells; and human CD43 was expressed on T cells, precursor B cells, activated B cells, natural killer (NK) cells and granulocytes. In our study, these markers were tested weekly after transplantation. CD3 and CD43 levels were higher in the piPSC, ciPSC, niPSC, UCBC and hESC groups than in the α-MEM group (Fig. 4a), but no significant differences were observed among the experimental groups. For example, CD8, CD3 and CD45 (leukocytes); CD71 (immature red blood cells); CD235a (mature red blood cells); and CD31 and CD43 (other human-specific antibodies) were detected in the cell transplantation groups but not in the blank control group (Fig. 4b). In other words, after IVS-II-654 β-thalassaemia mice underwent nonmyeloablative treatment with busulfan and cyclophosphamide, stem cells derived from β-thalassaemia-iPSCs before and after repair, hESCs and cord blood can survive and differentiate in mice.

**Fig. 5:**
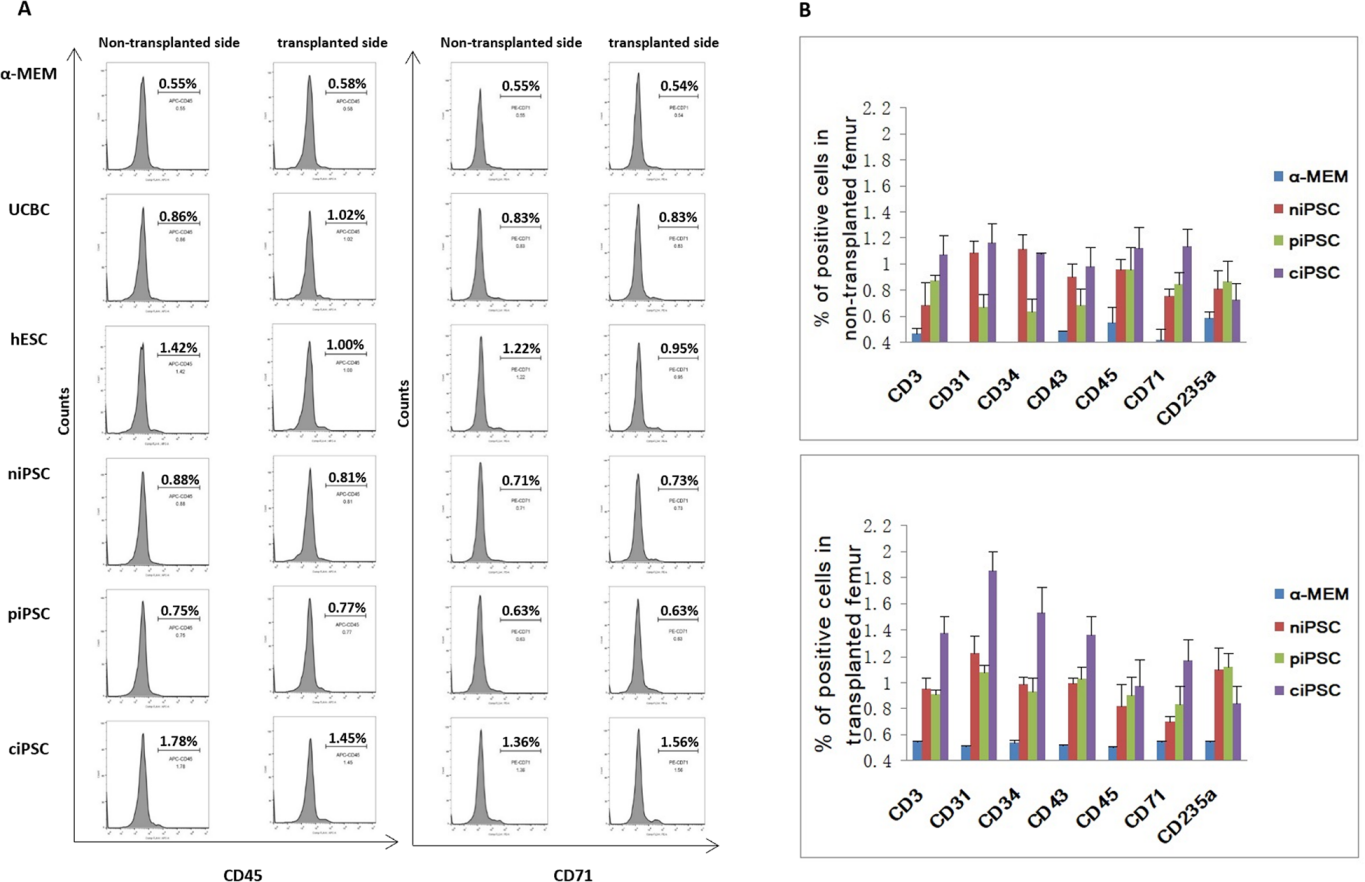
Haematopoietic differentiation of β-thalassaemia-iPSCs and genetically corrected iPSCs in BM after transplantation. **a** Flow cytometry analysis of human CD45 and CD71 in BM from the five groups of mice transplanted with (right panel)/without (left panel) α-MEM, UCBCs, hESCs, niPSCs, piPSCs or ciPSCs. **b** Flow cytometry analyses of human CD3, CD31, CD34, CD43, CD45, CD71 and CD235a in BM cells

To detect the differentiation ability of transplanted cells in BM from the left femur (transplantation) and right femur (no transplantation), all mice were sacrificed 10 weeks after cell transplantation. BM cells were collected from the transplantation (left) femur and nontransplantation (right) femur, and human CD3, CD31, CD34, CD43, CD45, CD71 and CD235a were detected by flow cytometry. CD45 and CD71 were detected in similar proportions in the transplantation and nontransplantation femurs from each group (Fig. 5a). The levels of these CD markers were higher in the piPSC, ciPSC, niPSC, UCBC and hESC groups than in the control group injected with α-MEM (Fig. 5b). The expression of human-related markers was detected in the nontransplantation mouse femur, indicating that human HSCs derived from iPSCs and UCBCs can successfully home in mice.

### Pathological results

**Fig. 6:**
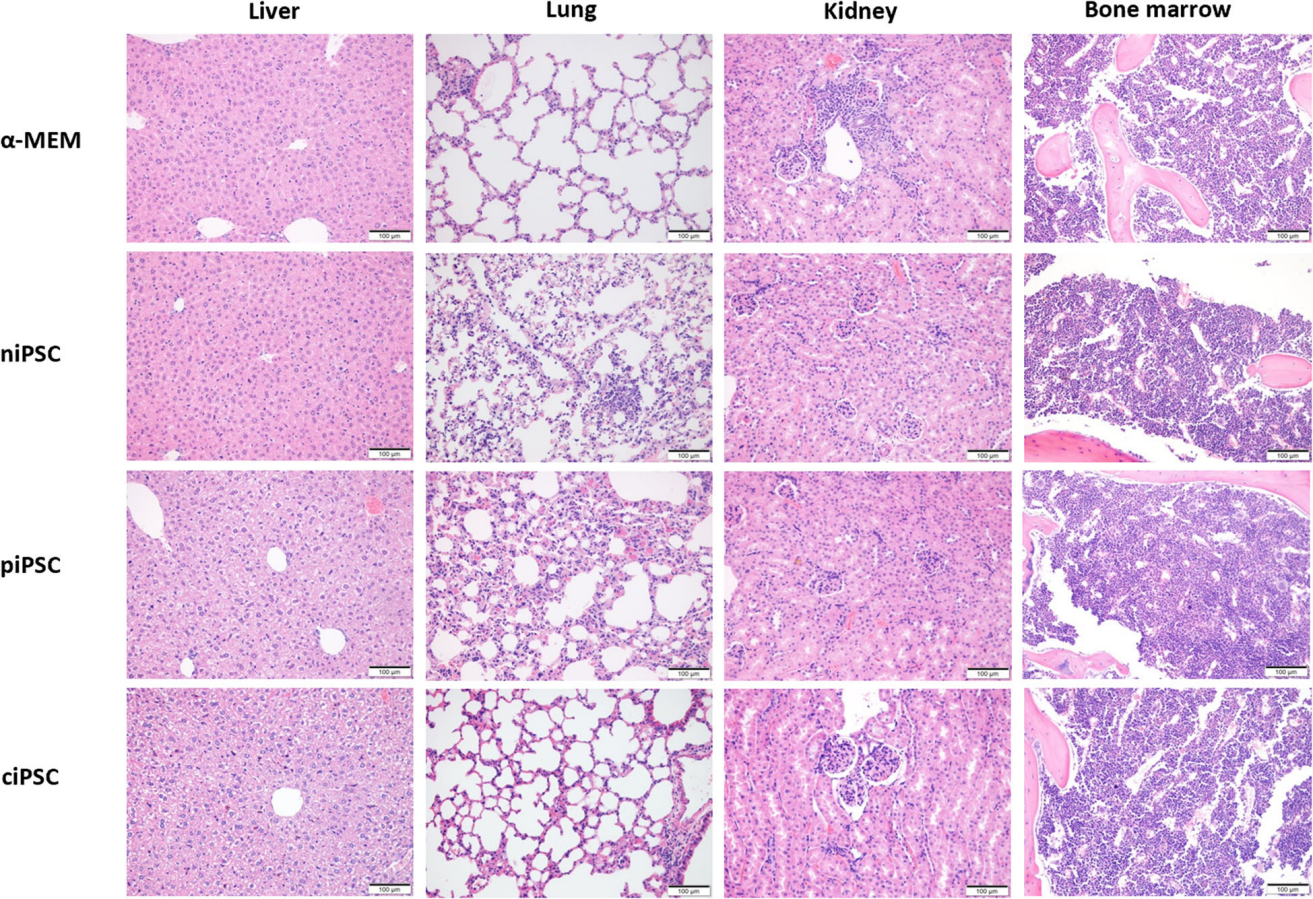
Pathological results of the mouse liver, lungs, kidneys and BM after transplantation. No tumours were observed in the liver, lungs, kidneys or BM of mice in any group at 10 weeks after transplantation. Scale bar, 100 μm

The potential tumourigenicity of iPSCs is an important factor in their clinical development. Therefore, we determined by histopathology whether tumour formation occurs in mice that undergo BM transplantation. In fact, no tumours were observed 10 weeks after transplantation in the liver, lungs, kidneys or BM of mice in each group (Fig. 6). Therefore, we believe that iPSC-derived HSCs have no short-term tumourigenic effect in mice, and long-term observation may be needed in future studies to confirm that genetically corrected iPSC-derived HSCs are safe for use in humans.

## Discussion

Thalassaemia is the most common genetic disease in the world and is characterized by genetic defects in HBB synthesis. Patients with severe forms of the disease require life-long blood transfusions and iron chelation therapy. A clear and effective way to treat thalassaemia is allogeneic HSC transplantation, but the use of this method is limited by the lack of HLA-matched donors and the risk of post-transplant complications, such as graft failure and graft-versus-host disease (GVHD) [20]. Because iPSCs come from patients themselves, their use avoids the ethical problems associated with ESCs and is associated with relatively little immune rejection. iPSCs are considered the most promising tools for treating β-thalassaemia with gene repair technology by inducing their haematopoietic differentiation into HSCs and transplanting them into patients.

In recent years, the rapid development of iPSC technology has provided an in vitro culture strategy to obtain pluripotent stem cells derived from patients’ own somatic cells for use in regenerative medicine. This approach avoids not only the problem of autoimmune rejection but also ethical problems. Researchers in our laboratory and others have shown that HBB production by HSCs can be restored by genetic correction of patient-specific iPSCs [14, 19, 21–24]. However, many obstacles must be overcome before iPSCs can be clinically applied, including determining the subsequent impact of establishing iPSCs in patients, whether HSCs survive and differentiate in vivo after genetic correction and whether HSC transplantation lead to tumour formation. The commercial use of the Sendai virus, which expresses four “Yamanaka” reprogramming factors, in the iPSC approach has enabled the widespread use of iPSC technology and represents a notable step towards clinical application [25, 26]. The development of clustered regular interval short palindromic repeat (CRISPR) systems has generated a powerful tool for targeted genetic modification and more convenient genetic repair [27]. Therefore, we focused on the differentiation of HSCs derived from ciPSCs in vivo in a thalassaemia mouse model. This report describes our first attempt to directly transplant gene-corrected iPSC-derived HSCs into the B6;129P2-*Hbb*^*tm2Unc*^/J thalassaemia mouse model. Our study advances the field by demonstrating the potential application of gene therapy with iPSC-derived HSCs and helps establish a new avenue for the clinical treatment of β-thalassaemia major.

Traditional pretreatment for HSC transplantation mainly involves a lethal dose of a myeloablative preconditioning agent. This method of immunosuppression is robust and has a strong killing effect on leukaemia and tumour cells [28, 29]. However, further research revealed that pretreatment at a lethal dose inevitably leads to pretreatment-related events, such as infection, haemorrhage and even death, due to the clearance of the haematopoietic system from the BM. The mortality associated with transplantation preconditioning can be 10~20% or even greater [30]. At present, the majority of animal models used in basic research use TBI [31] as pretreatment prior to transplantation. However, since the early 1980s, TBI prior to human HSC transplantation has been gradually replaced in clinical practice by combinations of chemotherapeutic drugs, such as busulfan and cyclophosphamide, due to the limited use of radiation sources in many laboratories. Busulfan and cyclophosphamide, when used in combination to clear noncirculating primitive stem cells, have similar effects as TBI [15, 16, 32], but the appropriate dosages of these drugs are not clear. Therefore, as the nonmyeloablative effect of the chemicals used to pretreat the HSCs before transplantation is similar to that of TBI, we further investigated the appropriate dosages of the nonmyeloablative drugs in this study. All mice in group 35, which were treated with 35 mg/(kg day) busulfan, died during drug treatment, suggesting that the dosage was too high and thus killed all the mice. Our results indicated that 25 mg/kg busulfan combined with 50 mg/(kg day) cyclophosphamide for 6 days was an ideal nonmyeloablative preconditioning regimen for transplantation therapy in β-thalassaemia mice with possible applications in the clinic.

In this study, we used a thalassaemic mouse model to mimic thalassaemic patients. As there is currently no animal model of thalassaemia and immunodeficiency that allows cell implantation, the concurrent use of drugs that stimulate transplanted cell growth after nonmyeloablative treatment is required to achieve immunosuppression in the immunocompetent thalassaemia mouse model. This mouse has the typical symptoms of thalassaemia but normal immune function. Routine blood test results showed that HB and erythrocyte levels in peripheral blood decreased greatly after drug treatment. After 2 weeks of pretreatment and transplantation, the two indicators began to increase in mice in each group, but no significant differences were observed among groups. It may be that the model mice were not completely immunosuppressed and maintained a certain level of immunity against transplanted material, meaning that the implants could only be maintained within a certain small range; this may cause the number of cells in each experimental group to remain low and not differ among groups. To confirm haematopoietic function in thalassaemic mice, we determined the extent of haematopoietic function of the graft and self-recovery and detected human markers (CD3, CD45, CD71, CD31, CD34, CD43 and CD235a) in BM cells from both the transplantation and nontransplantation femurs; moreover, we confirmed that iPSC-derived HSCs can survive, home and differentiate in these mice. Although HSC homing is an accepted fact and intravenous injection protocols have been published, we aimed to reduce the loss of cells in blood circulation and increase the number of cells that reached the desired location; thus, we performed femoral injections to confirm whether the injected cells can be detected in the body at sites other than the injection site and in other bones. Similar to the results in a previous study that genetically modified iPSC-derived HSCs positively stimulate haematopoiesis in irradiated SCID mice [19], the haematopoietic results of our study suggest that genetically corrected iPSC-derived HECs successfully stimulate haematopoiesis in B6;129P2-*Hbb*^*tm2Unc*^/J mice after appropriate nonmyeloablative treatment. This study represents a novel, valuable attempt to determine whether these genetically corrected iPSCs can give rise to haematopoietic progenitors that undergo normal haematopoiesis in a β-thalassaemia mouse model.

However, the mechanism of stimulation in this study may be different. Our mice have normal immune function, and their own haematopoietic function will self-recover over time. Furthermore, transplanted cells may be killed by NK cells in these mice. These specifics may explain why HB levels and the RBC count increased quickly but remained below the normal baseline values. Regarding the number of cells transplanted, we referred to previous studies; when 5 × 10^5^ CD34^+^ HECs were transplanted into the mouse tibia, cell implantation and growth were detected [19]. Although we could not definitely determine whether the lack of detectable tumour formation is due to transplanted cell rejection, we performed relevant pathological tests to determine whether tumours had formed. Our results verified that gene therapy with transplanted HSCs established haematopoiesis in thalassaemia mice, further addressing the issues that prevent effective HSC transplantation in humans. Moreover, flow cytometry analysis of the differentiation abilities of erythrocytes and other blood cells suggested that the transplanted cells were functional and capable of undergoing haematopoiesis in thalassaemia mice, which is consistent with the results of previous in vivo studies [17].

In summary, we demonstrated that HSCs obtained from genetically corrected iPSCs can survive and differentiate into erythrocytes in mice that underwent nonmyeloablative preconditioning. In addition, we did not observe tumour formation in thalassaemic mice after transplantation. However, our results showed that cell implantation did not achieve the expected therapeutic effect (anaemia was not effectively treated). Considering that our mouse model has anaemia but otherwise exhibits normal function, it will significantly reject foreign grafts. Therefore, the implantation effect will be obscured by self-recovery, leading to anaemia symptoms that cannot be corrected. This effect may be the main reason that we successfully recovered haematopoietic function without observing significant differences between the experimental and control groups. Therefore, we propose administering an anti-rejection drug in combination with genetically modified iPSC-derived HSC transplantation; such an approach may be an important topic of our future research, which may bring us closer to human clinical translation.

## Conclusions

In conclusion, our study provides strong evidence that iPSC technology combined with gene targeting could potentially be used to treat a variety of human diseases, including genetic diseases such as β-thalassaemia major.

## Data Availability

Not applicable.

## Abbreviations

α-MEM: Alpha minimum essential medium
BMP4: Bone morphogenetic protein 4
CFU: Colony forming unit
CRISPR: Clustered regular interval short palindromic repeats
FBS: Foetal bovine serum
Flt3: Fms-related tyrosine kinase
GVHD: Graft-versus-host disease
HB: Haemoglobin
HSC: Haematopoietic stem cell
HSPCs: Haematopoietic stem/progenitor cells
IL-3: Interleukin-3
iPSC: Induced pluripotent stem cell
MACS: Magnetic-activated cell sorting
rhG-CSF: Recombinant human granulocyte colony-stimulating factor
rhEPO: Recombinant human erythropoietin
UCBC: Umbilical cord blood cell
VEGF: Vascular endothelial growth factor
WBCs: White blood cells
